# Residual Bioprosthetic Valve Immunogenicity: Forgotten, Not Lost

**DOI:** 10.3389/fcvm.2021.760635

**Published:** 2022-01-04

**Authors:** Paul Human, Deon Bezuidenhout, Elena Aikawa, Peter Zilla

**Affiliations:** ^1^Chris Barnard Division of Cardiothoracic Surgery, University of Cape Town and Groote Schuur Hospital, Cape Town, South Africa; ^2^Cardiovascular Research Unit, Faculty of Health Sciences, University of Cape Town, Cape Town, South Africa; ^3^Division of Cardiovascular Medicine, Brigham and Women's Hospital and Harvard Medical School, Boston, MA, United States; ^4^Faculty of Health Sciences, Cape Heart Institute, University of Cape Town, Cape Town, South Africa

**Keywords:** bioprosthetic, valve, extracellular matrix, decellularization, immunogenicity, pathology, inflammation, calcification

## Abstract

Despite early realization of the need to control inherent immunogenicity of bioprosthetic replacement heart valves and thereby mitigate the ensuing host response and its associated pathology, including dystrophic calcification, the problem remains unresolved to this day. Concerns over mechanical stiffness associated with prerequisite high cross-link density to effect abrogation of this response, together with the insinuated role of leaching glutaraldehyde monomer in subsequent dystrophic mineralization, have understandably introduced compromises. These have become so entrenched as a benchmark standard that residual immunogenicity of the extracellular matrix has seemingly been relegated to a very subordinate role. Instead, focus has shifted toward the removal of cellular compartment antigens renowned for their implication in the failure of vascularized organ xenotransplants. While decellularization certainly offers advantages, this review aims to refocus attention on the unresolved matter of the host response to the extracellular matrix. Furthermore, by implicating remnant immune and inflammatory processes to bioprosthetic valve pathology, including pannus overgrowth and mineralization, the validity of a preeminent focus on decellularization, in the context of inefficient antigen and possible residual microbial remnant removal, is questioned.

## Introduction

Despite deployment of valve heterografts as native valve replacement prostheses more than a half century ago (1), understanding of the specific mechanisms involved in the most pernicious of observed xenogeneic valve substitute pathologies, namely dystrophic mineralization, remains elusive to this day. Remarkably, none of the initial grafts implanted, prepared either by chemical sterilization or by formalin treatment and retrieved after 3 months to 3 years, demonstrated any evidence of mineralization but did exhibit moderate inflammatory infiltrates (2). The authors of that pioneering study quickly recognized that the observed host response was indicative of persistent antigenicity in the heterograft tissue and elimination of antigenic components was highlighted as one of the key criteria for ensuring graft longevity. With respect to the use of formalin, it was also recognized that both its susceptibility for cross-link reversibility and its inefficacy in eliminating glycoprotein and collagen non-helical terminal telopeptide antigenicity detracted from its potential use. The bifunctional dialdehyde glutaraldehyde (GA) (0.65%; w:v) was identified as a suitable alternative, together with periodate oxidation to create zero-length cross-links with structural glycoproteins (2). These innovations were ground-breaking, arriving within 4 years of the initial implants but, apart from decellularization and the introduction of variations of cross-link chemistries deployed in contemporary counterparts, in general remain little changed.

## Distraction, Division, and Decellularization

Due to the quest for organ donor alternatives and the experiences with solid organ xenografts, which highlighted the catastrophic phenomenon of hyperacute rejection, attention has recently refocused on the cell surface molecule galactose-α-1,3-galactose (α-Gal) found in New World monkeys and candidate non-primate mammal donors (3) such as the pig, cow and sheep. This molecule was identified as the target for preformed, naturally occurring, antibodies (PFNAb) found in humans and Old World primates, such as the baboon and rhesus and vervet monkeys, and was quickly labeled as the principal “xeno-antigen.” This preoccupation, together with the assumption that the problem of extracellular matrix (ECM) antigenicity in bioprosthetic valve tissue was satisfactorily resolved through chemical cross-linking, appeared to translate into the belief that persistent valve pathologies could be ascribed entirely to the presence of donor cells found in tissue valve heterografts, and that remnant immunogenicity could simply be addressed by cell removal alone.

Ironically, evidence has been presented which suggested that myocardial, but not valvular, tissue in the domestic pig expressed the α-Gal epitope and, in a pig to primate xenotransplant model, unfixed valvular tissue was seen to be immunologically privileged (4). However, since hyperacute rejection rapidly occurred due to microvascular binding of IgM and complement, the exact long-time outcome potential of porcine valvular tissue could not be confirmed. Despite this, the suggestion to use unfixed porcine valves for valve replacement as an alternative to mechanical and chemically treated bioprosthetic valves was proffered (5). In contrast, evidence of the mineralization of unfixed, acellular, pig and kangaroo tissue, implanted in sheep, both confirmed the immunogenicity of decellularized tissue and the role thereof in mineralization (6).

A study which evaluated the subdermal *in vivo* response in juvenile rats to glutaraldehyde (0.6%; w:v) cross-linked pericardial tissue from α-Gal knockout pigs, determined calcium levels to be significantly lower than in the corresponding wild-type equivalent after 1 month (7). Moreover, detergent-treated and cross-linked wild-type pericardium, arguably therefore decellularized, resulted in negligible levels of mineralization but which were again significantly increased following pre-incubation with purified human anti-α-Gal antibody. This not only confirmed the role of tissue-specific immunoglobulin in accelerating calcification of bioprosthetic pericardial tissue in accordance with our rabbit serum pre-incubation study (8), but also highlighted the problem of insufficient washout of xeno-antigens following detergent treatment. In that study, where porcine aortic wall implants were pre-incubated with sera obtained from rabbits immunized with extracts of the same tissue and implanted sub-dermally, tissue-specific immunoglobulin would undoubtedly not have been directed at the α-Gal epitope, given the rabbit recipients were α-Gal positive and, thereby, anti-α-Gal negative.

Detection of α-Gal based on lectin binding is common practice but is questionable in detergent-treated tissue due to its specificity for non-α-Gal carbohydrate structures likely to be exposed through that denaturing process (9). Development of the M86 monoclonal antibody and its application in a quantitative enzyme linked immunosorbent assay (ELISA) assay (10) aided in providing more definitive evidence of the distribution of the α-Gal epitope in porcine (11) and bovine (3) tissues, although lack of evidence for its presence in heart valve tissue was conspicuously absent. It is, however, apparent, based on monoclonal anti-α-Gal immunostaining and more quantitative studies, that porcine valvular tissue appears to contain at least a modicum, if not more, of α-Gal epitopes, albeit less in aortic compared to pulmonary tissue (9). It is difficult to discern whether this extends beyond the vascular endothelium to stromal tissue but, if predominant in the former, may be inconsequential or easily lost as a result of denudation of lumenal endothelium during commercial processing.

Additionally, not only does the α-Gal epitope appear resilient to both GA fixation and decellularization, but the cross-reactivity of anti-α-Gal PFNAb against so-called “competitor” epitopes, themselves equally pervasive, may also contribute to long-term failure (12). Naso et al. (13) have more recently provided evidence, based on an adapted M86 ELISA, of native and contemporary bioprosthetic valve construct tissues, including from the porcine, valve-based, Epic and bovine pericardial Mitroflow tissue valves, not only highlighting the presence of α-Gal in both native bovine and porcine tissue but, importantly, its diminution following commercial processing. The apparent elimination of this epitope in the Epic valve is, however, offset by its reported early mineralization (14). Furthermore, Moczar et al. (15) provided evidence of IgG and complement in 20/22 failed Mitroflow valves, despite the 75% reduction in α-Gal epitope reported by Naso et al. While we would concede that the presence of α-Gal, capable of eliciting a host response, may indeed contribute to ensuing pathology of the implanted tissue, it likely does not represent the quintessential xeno-antigen and its depletion should not be held as the sole benchmark for determining xeno-reactivity. Certainly, given the inherent resistance to adequately remove such immunogens from tissue where they exist, the promise may well lie in the use of purpose-bred knockout animals, particularly targeting not only α-Gal but the full “trifecta” of known xeno-antigens, including N-glycolylneuraminic acid (NeuGc) and the glycan products of beta-1,4-N-acetyl-galactosaminyl transferase 2 (16). While the cytidine monophosphate-N-acetyl-neuraminic acid hydroxylase enzyme involved in the synthesis of NeuGc is lacking in humans, it exists in other mammals, including non-human primates (17). Consequently in humans, as with α-Gal, preformed natural antibody to NeuGc potentially exists, likely due to dietary uptake of this antigen present in red meat and dietary products (17). It has been demonstrated in both porcine aortic valve leaflet and pericardium as well as in bovine pericardium (16, 18) with effective knockout of the respective enzyme and antigen genes abrogating human immunoglobulin binding *in vitro* (16, 19, 20).

Removal of α-Gal is not limited to knockout technology and, while GA treatment alone is purported to diminish the antigen exposure by half (21), enzymatic treatment with green coffee bean alpha-galactosidase (22), or a recombinant human equivalent (23), has been shown to effectively remove it completely. Kim et al. showed reduction, but not abolition, of calcium in GA cross-linked, decellularized and alpha-galactosidase treated bovine pericardium after 4 months in α-Gal knockout mice, designed to simulate the clinical scenario (24). It is likely that this was due to the presence of xeno-antigens other than α-Gal since calcium levels in concurrently implanted primate tissue were statistically equivalent. Therefore, enzymatic treatment offers an alternative to employment of α-Gal knockout donors with potential benefit but equally fails to represent a panacea for achieving immunologically inert bioprosthetic tissue.

Although work has focused on the development of transgenic pigs for solid organ xenotransplantation incorporating human decay accelerating factor, which promises to mitigate complement fixation (25) and other regulatory molecules, application of this technology is unlikely to benefit devitalised bioprosthetic valves. However, a preclinical heterotopic cardiac xenotransplant study in baboons (*Papio anubis*) involving donor pig hearts transgenic for human complement regulatory factors CD59 and decay accelerating factor demonstrated intact valve tissue and confirmed by immunohistology to be immunoglobulin free and with a confluent endothelium, despite failure of the myocardium itself after up to 11 days due to IgM binding and establishment of membrane attack complexes (5). Manji et al. postulate that, provided α-Gal and NeuGc are eliminated in knockout strains of donor pigs, transfer of human genes associated with regulation of complement may suffice to protect against the response to what they term “minor antigens” (26). They further highlighted the need for such genetically engineered bioprosthetic heart valves in children and young patients where structural valve destruction is likely linked to a xenograft-like rejection of the prosthesis (27).

Nevertheless, the enthusiastic push for a decellularized bioprosthetic tissue valve does hold merit. Firstly, it offers a potentially pristine, standardized, three-dimensional scaffold uncluttered with the detritus associated with the consequences of inevitable cell death due to a combination of processing artifacts, including warm ischaemia, hypotonic shock, autolysis, and microbial degeneration. Immediate processing combined with high GA concentrations offers reduced mineralization (28), which has been shown to be coincidental with improved ultrastructural preservation (29). Since these approaches, while ideal from a reduced immunogenicity perspective, are impractical due to logistical and aortic wall stiffness considerations, an acellular prosthesis is arguably preferable. The promise of repopulation of such material with host cells is tantalizing and holds great promise, especially in young children where a growing valve might avoid multiple valve replacement procedures during their lifespan (30), but is yet elusive and seemingly dependent on a macrophage driven response (31).

Despite some early successes (32), the perplexing question of whether inflammation is a prerequisite for repopulation remains, as does the matter of the suitability of the composition of that ingrowth when donor tissue is anatomically different from that of the intended target. For example, the use of pericardial tissue to replace the complex triple-layered design of tricuspid leaflets with the beneficial damping qualities of the spongiosa and the overdistention avoidance of the ventricularis, may be too ambitious, despite the successes of contemporary non-decellularized, cross-linked, pericardial valve constructs. Many of the reports suggestive of the repopulation potential of acellular xenogeneic tissue typically misinterpret an inflammatory infiltrate with “regenerative potential,” which is yet to be adequately defined. It is conceivable that inflammation may lay down some of the necessary “guide-rails” suited to subsequent infiltration and establishment of the correct connective tissue. However, the degradation products of an inflammatory response, especially when unresolved, will, through a process of defective phagocytosis, lead to pathological tissue remodeling, including apoptotic bodies, extracellular vesicles and other debris-all typical initiators of dystrophic calcification. Through mechanical forces exerted by the hydroxyapatite crystals on the scaffold, irreversible tissue disruption and delamination will be inevitable.

**Figure 1:**
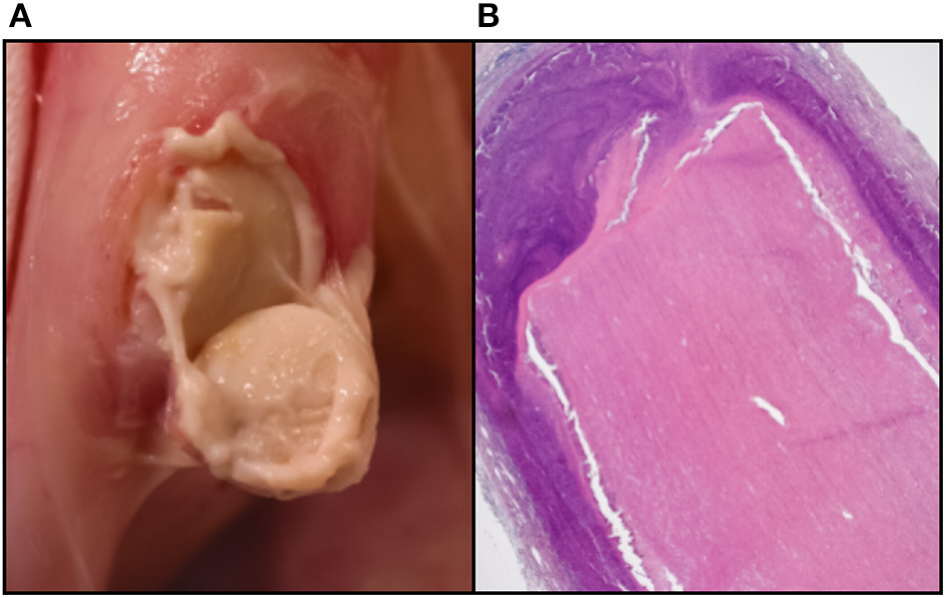
Sterile granulocytic pus response to SDS-based decellularization of porcine aortic wall in the rabbit subdermal model. **(A)** Macro photo at time of retrieval. **(B)** Haematoxylin & Eosin histology showing thick granulocytic rim surrounding the implant.

In addition, the complex mix of detergents, enzymes and inhibitors used in decellularization can themselves induce an immune response and care needs to be taken to adequately wash these from the tissue (33). In addition to the retention of these exogenous reagents, the incomplete washout of disrupted endogenous lipid membrane micelles, typically also associated with partially solubilized cellular antigens, which may have otherwise been less exposed, can induce a sterile granulocytic response with catastrophic consequences (34). Further evidence confirms that incomplete immunogen washout in porcine pulmonary valve tissue is capable of eliciting both a substantial foreign-body reaction on the luminal surface as well as a lymphoproliferative response, comprising T- and B-lymphocytes and plasma cells with evidence of marked immunoglobulin secretion, in children (35). We have observed a severe, sterile, pus-like granulocytic response to porcine aortic wall tissue decellularized using the anionic detergent Sodium dodecyl sulfate (SDS) in rabbit, rat, and primate (baboon and vervet) subdermal implant models, despite exhaustive washout (Figure 1).

SDS is a logical choice for ensuring thorough removal of cellular and non-structural proteins, as it impacts both cytoplasmic and nuclear membranes and results in extensive denaturation of tertiary and secondary conformation. However, it is equally capable of deleterious effects to collagen and elastin and thereby, not only altering the mechanical properties of the tissue but, also limiting the ingrowth potential of the tissue to allow for desirable cell repopulation (36). The bile acid/salt detergent, deoxycholate, offers advantages in solubilizing lipid membranes and being more compatible with the ECM, but their subsequent efficient washout can prove difficult. The non-ionic detergent, Triton-X, is less effective than SDS at cell component removal (37) and is facing a legislative ban due to ecotoxicity safety issues. Compared to others, SDS and deoxycholate were the only detergents capable of total removal of cells from porcine aortic leaflet tissue (38) with their combination being reported as optimally anti-inflammatory to macrophage and T-lymphocyte infiltration (37). In contrast, our experience with decellularized porcine and bovine pericardial tissue using a combination of Triton–X and sodium deoxycholate, suggested only scanty involvement of granulocytes, with the response involving predominantly a mixed macrophage/lymphocyte response after six-week subdermal implantation in the rat.

**Figure 2:**
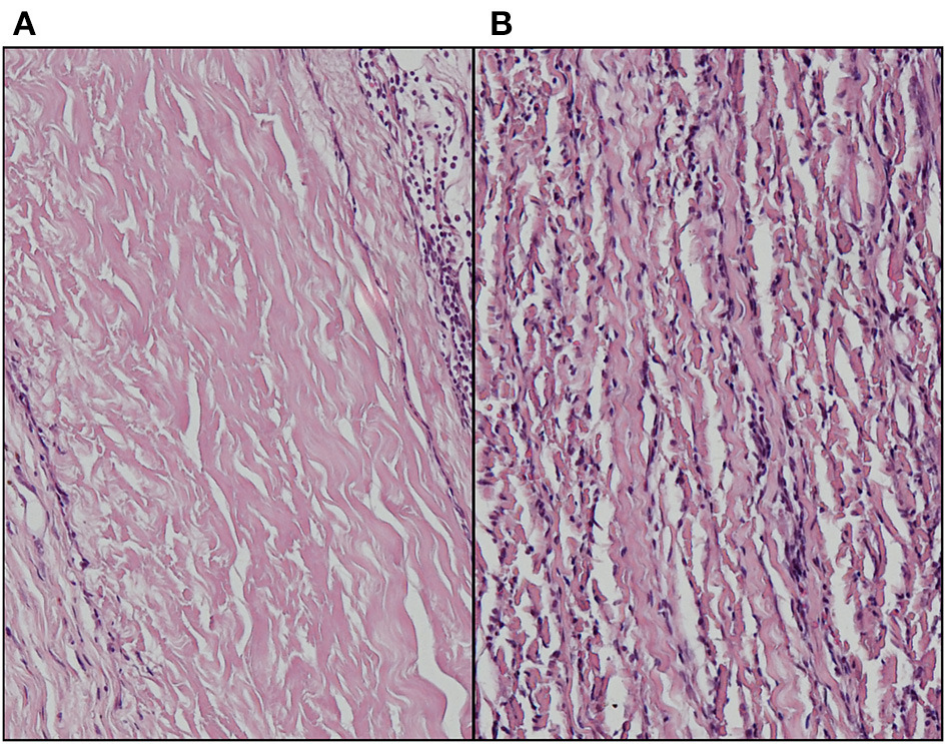
Absence of inflammatory infiltrate in **(A)** electrophoretically enhanced anionic detergent decellularization of bovine pericardium following 6-week subdermal implantation in the rat compared to **(B)** non-ionic decellularization with exhaustive washing (Haematoxylin & Eosin stain).

Enhanced methods of decellularization have been used to bridge the problem of inefficient washout of disrupted components, including vacuum assisted (39) and electrophoretic approaches (40, 41). Our own exploration into enhanced electrophoretic washout following decellularization using a semi-dry method following decellularization of bovine pericardium with SDS demonstrated negligible inflammatory infiltrates within the interior of the tissue but with a moderate rim of surface inflammatory cells in the subdermal rat model (Figure 2).

Accurate methods of validating the efficiency of decellularization are paramount and this, by necessity, requires a detailed knowledge of the identity of the immunogens present in the respective tissues. Quantitative DNA analysis, for example, has been shown to be a poor litmus test for residual immunogenicity (42) and evaluating extractable soluble protein provides little assurance of the removal of lipid soluble ones. Other structural proteins, such as fibronectin mentioned above, and which appear resilient to extraction or indeed adequate cross-linking, are highly immunogenic (43).

Clearly, a “marriage of convenience” between decellularization and chemical cross-linking is required to adequately address residual immunogenicity, especially of the ECM. Despite concerns to the contrary, the use of GA, long thought to promote bioprosthetic mineralization, as an adjunct to decellularization of bovine pericardium, failed to undo the anti-calcification benefit of decellularization in our hands (44). Whether this suggests that leaching of monomeric GA is diminished in acellular tissue, or perhaps is confounded by the presence of the cellular compartment in intact tissue, or both, remains to be determined.

## Extracellular Matrix Immunogenicity

We had previously demonstrated in a subdermal rabbit model that residual immunogenicity of porcine aortic wall led to an acquired humoral immune response in tissue fixed with a GA concentration applied commercially (45). Immunoglobulin specific to porcine fibronectin, a structural glycoprotein known to associate with collagen in the ECM and distinctly present in the basement membrane and therefore principally exposed in denuded bioprosthetic valves, persisted in rabbits immunized with tissue cross-linked with a range of GA concentrations spanning and far exceeding those used commercially. It was only abrogated when cross-links were further extended between free aldehyde groups using the diamine L-Lysine, which was confirmed to increase shrink temperature and therefore, hypothetically, cross-link density (43). Decellularization of bovine pericardium, a tissue widely used in minimally invasive transcatheter aortic valve implantation, has demonstrated a resilience in the removal of this structural glycoprotein, despite diminishment of other ECM components collagen IV and laminin (46), suggesting that both cell removal techniques and commercial cross-linking practices may likely fail to fully abolish this tenacious xeno-antigen.

**Figure 3:**
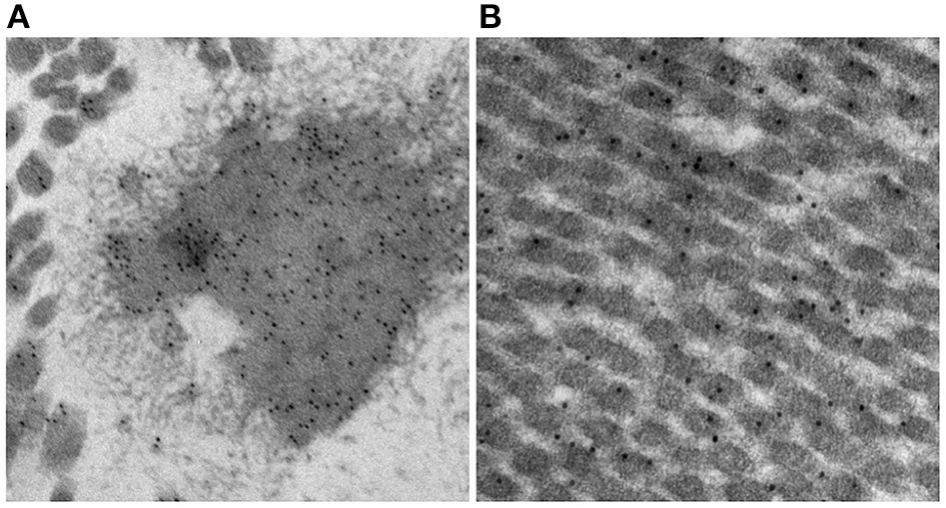
*In vitro* evidence of post-implant rabbit sera specificity to elastin **(A)** and collagen **(B)** in GA fixed (0.2%) porcine aortic wall using gold-labeled anti-IgG.

There are mixed reports regarding immunogenicity of the principal structural components of the ECM, namely collagen and elastin. Bayrak et al. report an absence of *in vitro* evidence for dendritic cell maturation or B- and T-lymphocyte response to bovine and porcine type 1 collagen and elastin (47). In contrast, Biermann et al. report acute T-lymphocyte infiltration in acellular, unfixed, cryopreserved porcine pulmonary valve xenografts implanted in sheep (48). While anecdotal, we have observed *in vitro* evidence for post-implant rabbit IgG binding under transmission electron microscopy to both collagen and elastin in 0.2% GA fixed porcine aortic wall using gold-labeled anti-IgG (Figure 3). The calcium observed by Van Nooten et al. in acellular pig and kangaroo aortic valves after implantation in sheep for 4 months was seen to be in close association with collagen fibers while non-decellularized porcine valves failed to calcify (6). Electrophoretic analysis of decellularized porcine valve tissue confirmed the presence of residual proteins and proteoglycans distinct from those found in decellularized human valves, with persistent mononuclear infiltration in the former but which was completely eliminated in the latter (49).

Additional data on the immunogenicity of proteoglycans is scanty, although *in vitro* co-culture experiments using peripheral blood mononuclear cells with electrospun decorin was indicative of low immunogenicity (50).

Demonstration of the undisputed role of bioprosthesis-specific immunoglobulin as a key contributor of subsequent mineralisation (8), and the recent finding that calcification and degeneration of GA-fixed porcine bioprosthetic tissue in a macrophage-depleted mouse model were decreased (51) are equally noteworthy in highlighting the absolute requirement to diminish residual immunogenicity as a means of increasing longevity of bioprosthetic valves, especially in children.

## Immunogenicity and Phylogenetic Disparity

It is undisputed that, apart from the inflammatory response associated with surgical trauma and the response to synthetic material such as valve skirts and sutures, transplantation of viable valve tissue between syngeneic donor/recipient pairs is intrinsically successful and typically requires minimal or no immunosuppression. This is also clinically evident from the realization that the longevity of conjoined homozygous twins remains unchallenged, at least from an immunological perspective. The approach is understandably cautious when transplantation of solid organs is performed between dizygotic twins or between closely matched individuals despite zero-HLA-mismatch, with low-level immunosuppressive regimens still being advocated (52), although successful outcomes following renal transplantation between homozygous pairs have been reported without continuous immunotherapy (53, 54), but long-term studies are lacking.

Pulmonary autografts represent the perfect matching tissue for valve replacement, typically used in the aortic (Ross procedure) or even the mitral position (55). Seven-year follow-up of the latter confirmed non-calcified leaflets. In Ross procedure patients, the limited ability of the wall of a living autograft root to adapt to the demands of the aortic position may sometimes overshadow its success (56). Yet, most importantly, remodeling has been reported in the absence of inflammation (57, 58).

Allogeneic (homograft) valves, on the other hand, have been reported to be associated with both post-operative fever and valvular dysfunction, which correlated with the extent of MHC Class 2 HLA-DR mismatches (59). Indeed, experimentally, cytotoxic alloreactive T-cell involvement has been implicated in the thickening and destruction of allogeneic leaflets in valves implanted infra-renally in rats compared to syngeneic and T-cell deficient animals (60). Clinically, in a small retrospective study, five infants, but none of the adults, who underwent aortic valve replacement with homograft valves and which failed within 8 months, all exhibited distinct T- and, in three cases, B-lymphocytic infiltrates (61). Curiously, only the adults, as well as a 13-month-old child showed evidence of calcium, despite the absence of inflammatory cells.

One of the original reports in 1996, which hinted at an immune mechanism underlying dystrophic calcification of bioprosthetic tissue, evaluated the outcome of a small cohort (eight GA-fixed bovine pericardial and two GA-fixed porcine aortic valve prostheses) of aortic valve replacement patients with aortitis, and who had received a long-term regimen of steroid therapy (62). Despite a mean follow-up exceeding 11 years, no re-operations were required for stenosis of the replacement prosthesis, and mineralization was “minimal” in three of the pericardial valves requiring replacement.

The success or failure of solid organ xenotransplantation was loosely categorized by Calne as either “concordant” or “discordant,” implying potential success or potential failure dependent on phylogenetic heterogeneity between donor and recipient (63). Using a heterotopic cervical cardiac xenotransplant model in the Chacma Baboon (*Papio ursinus*), we were able to demonstrate distinct histopathological differences associated with increasing genetic disparity between donor and recipient (64). The response to Vervet Monkey (*Cercopithecus aethiops*) myocardium was partially analogous to allogeneic transplant pathology, being mainly mononuclear with aggregations of T-lymphocytes. Rejection episodes were successfully treated with short duration prednisolone rescue therapy but presented with additional features of vascular rejection (interstitial hemorrhage, oedema, and myocyte necrosis). This, however, differed from the histopathological features associated with full-blown hyperacute rejection, typical of pig to baboon transplantation of vascular organs, which included vascular microthrombi, worsened oedema and hemorrhage and rapid vascular congestion and cyanosis, but without the cellular infiltrates. Nevertheless, while the cellular response could be averted with treatment in the vervet to baboon model, the humoral response eventually dominated, leading to inevitable failure. The potential survival times of such “concordant” grafts inversely correlated with titres of PFNAb, suggesting that success could be more accurately and definitively ascertained by their relative titer than the genetic donor-recipient disparity. Clearly, however, a widely divergent pair, such as the pig and baboon combination, might arguably be more accurately classified as “discordant” without knowledge of specific titer, given the higher likelihood of a lack of phylogenetic conservation. Inevitably, even with ingenious attempts at minimizing the deleterious effects of PFNAbs, which offer short-term success (65), all forms of cross-species transplantation are potentially unsuccessful without genetic engineering approaches to avoid known immunogens.

The same principal likely applies to devitalized tissues used in the fabrication of bioprosthetic valves. Certainly, donor and recipient choice was demonstrated by Carpentier et al. (66) to affect levels of subsequent calcification following subcutaneous implantation in a variety of species. However, despite recognition of the role played by graft immunogenicity in the ensuing inflammatory response, the same connection was not assumed with respect to the process of mineralization. Furthermore, a realization of the interrelationship between donor and recipient was not apparent. Logic might be expected to predict the pig to be most related to the cow but less so to the chicken. However, calcium levels in porcine cusps were significantly higher when implanted in the rabbit and rat, compared to the cow and hen, which showed equally negligible levels. Phylogenetic disparity needs to be taken into context, therefore, with respect to the conservation of specific epitopes present in the tissue in question, rather than donor-recipient evolutionary distance. The specific realization of the conservation of the α-Gal antigen in pigs, but not in humans therefore, together with the coincidental existence of PFNAb in the latter, likely directed toward a microbial epitope but capable of cross-reactivity with α-Gal, is critical in predicting outcome.

NeuGc is a case in point, occurring not only in potentially discordant donors such as the pig, but in the more otherwise phylogenetically closer non-human primate. It's relevant absence in corneal endothelial cells and pancreatic islets in wild-type pigs suggests use of NeuGc knockout donors (or combined with α-Gal knockout) of corneal or pancreatic tissue to be inappropriate compared to heart and aortic valves, for example, where significant reduction of the antigen holds much promise (19). While bioprosthetic valves, by their non-vascularized nature, are not at risk for hyperacute rejection, the opsonisation thereof by immunoglobulin directed at xenoantigen, whether α-Gal, NeuGc, fibronectin, or other residual or insufficiently masked epitope, will fix complement and/or induce macrophage and polymorphonuclear neutrophil adhesion with subsequent infiltration and phagocytosis or latent damage through frustrated phagocytosis and the formation of foreign body giant cells. This often insidious process, which continues beyond an early acute lymphocyte phase (67), underlies the importance of ensuring complete abrogation of tissue immunogenicity, either through complete removal (decellularization with extensive washing or enhanced electrophoretic removal), modification (through epitope capping) or masking (chemical cross-linking) of xeno-antigens.

With regard to the latter, we have advocated an engineered approach toward cross-linking decellularized xenogenic pericardium with GA, specifically through incorporation of extender diamine molecules, such as L-Lysine, and reduction of labile Schiff bases to avoid monomer leaching (44) which may again lead to exposure of epitopes over time. While GA remains the principal cross-linking agent used in contemporary bioprosthetic valves, alternatives, including genipin, which has shown potential for reduction of calcification (68), have been explored. However, decellularized porcine aortic valve scaffolds cross-linked with genipin and implanted in the pulmonary position of sheep for 6 weeks, while displaying significantly attenuated inflammatory infiltration, demonstrated persistent immunoglobulin binding (69).

## Microbial Contamination

**Figure 4:**
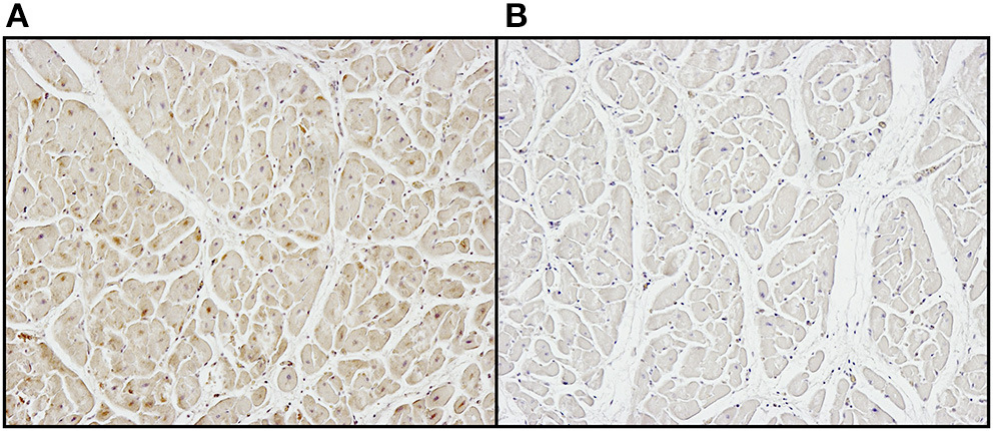
Positive immunostaining **(A)** to Gram+ bacteria in unimplanted commercial bioprosthetic valve tissue compared to **(B)** negative control.

**Figure 5:**
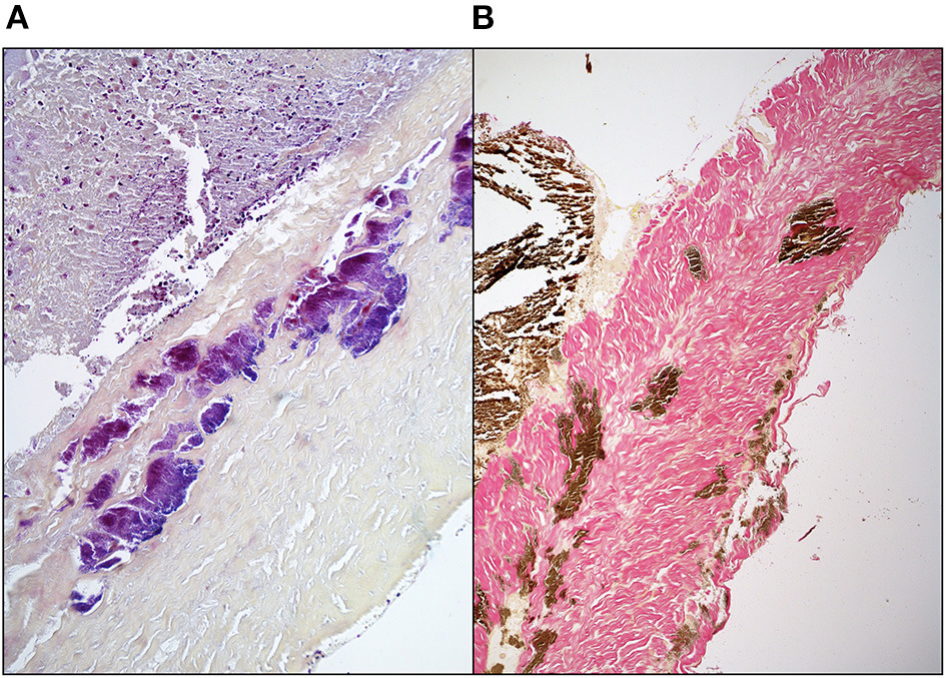
Suspected colocalization of areas of Gram + bacterial contamination of GA-fixed bovine pericardial tissue and mineralization in a stented mitral valve replacement bioprosthesis retrieved after 112 days **(A)** Brown & Brenn stain **(B)** Von Kossa & Van Gieson stain.

**Figure 6:**
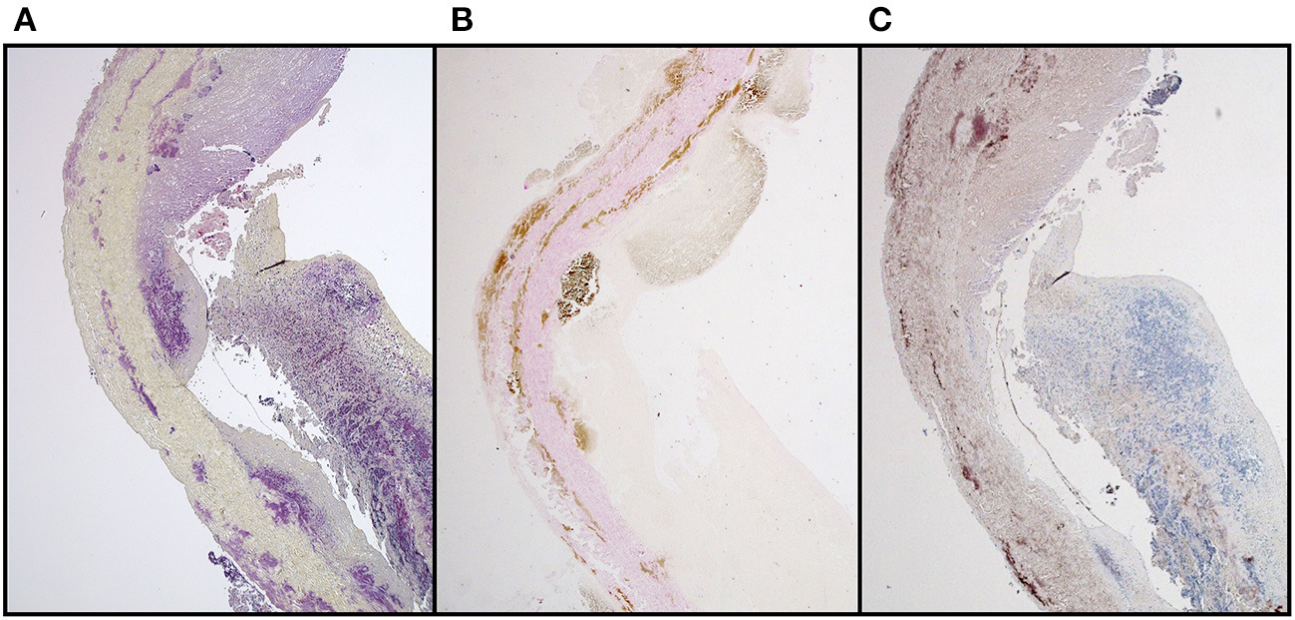
IgG binding in areas of Gram-positive bacterial contamination together with mineralization in a stented, GA-fixed, mitral valve pericardial bioprosthesis retrieved after 112 days **(A)** Brown & Brenn stain **(B)** Von Kossa & Van Gieson stain **(C)** Anti-Sheep IgG.

While it is not the intent to suggest that commercial bioprosthetic valves contain viable microorganisms, one must assume that microbial remnants could likely be contained therein. Whereas, abattoirs are not unclean, they are decidedly not sterile environments and contamination, either from the slaughtered animal itself, or from the processing area, is inevitable. Although the problem of porcine endogenous retroviruses is well-realized, especially in the field of xenotransplantation (70), bacteria may additionally contribute to bioprosthesis pathology. Other than gut and skin flora, endogenous bacteria too are known to exist in domestic pigs and may be associated with a foreign body response and/or dystrophic mineralization (71). Furthermore, tissue used in the fabrication of bioprosthesis typically remains in cold saline, potentially up to 48 h before being processed, which may permit colony expansion. Decellularization and/or GA fixation may remove the likelihood of transfer of viable endogenous bacteria or viruses to the human recipient, but remnants of these organisms will likely persist. We have certainly observed positive immunostaining in un-implanted commercial valve tissue using a monoclonal antibody specific for Gram-positive (Gram+) bacteria (Figure 4). In cases of contamination of bovine pericardial valves in the mitral position, colocalization between areas of positive Gram+ staining and positive Von Kossa staining depicting calcium deposits (Figure 5) and positive immunoglobulin G staining was observed (Figure 6). Contamination with Gram-negative bacteria or fungi was not associated with calcium deposits, suggesting an interplay between Gram+ bacteria, humoral immune response, and dystrophic mineralization. Mineralization of passenger bacteria in GA fixed tissue may also rely on an “inhibitor exclusion” mechanism whereby Fetuin, normally an inhibitor of crystal formation, is size-excluded from the Gram+ cell wall as well as additionally promoting their phagocytosis by neutrophils and macrophages (72, 73). This is highly speculative with respect to mineralization of bioprosthetic tissue, but the evidence of the pro-calcific nature of a yet unidentified, Gram + bacterium, is worthy of further investigation.

Certainly, while it is arguable that sterilization and cross-linking methods adequately render tissues used in the construction of bioprosthetic valves, free of any viable microbial organisms, further studies will be required to assess the relevant role of residual contaminants. Contemporary GA concentrations are incapable of total inactivation of bacterial spores, but added bioburden reduction strategies using alcohol, gamma radiation and other proprietary protocols are already unquestionably efficient at removing such a microbial risk. Should bacterial remnants, however, indeed represent initiation points for inflammatory migration and hydroxyapatite crystal growth, methods would need to be developed to limit or exclude these from the tissue. Prevention in the abattoir is unlikely, given strict controls disallowing chemical reagents at these locations. Shortening of the delay to processing is equally limited by the logistics of the expertise required for dissection and cleaning of valves and pericardial sacks. It would seem, therefore, that improved methods of decellularization and efficient washout, for acellular valves at least, offers the best hope for microbial antigenic determinant removal.

## Inflammation and Pannus Overgrowth

Intimal hyperplasia, encouraged by smooth muscle cell migration through severed elastic lamellae during the surgical implantation of freestyle aortic roots is also seen to occur in transcatheter aortic valve implantation cases (74) where there is no such trauma, and which suggests a different underlying mechanism. Furthermore, while a foreign body response to synthetic components has been shown to provide a sustained myointimal stimulus (75), the involvement of macrophages in inducing myointimal proliferation in transplant recipients (76) suggests that the same may be true for opsonisation of bioprosthetic tissue through immunoglobulin binding. Macrophage and foreign body giant cell formation are hallmarks of GA-fixed bioprosthetic heart valve pathology, and we have observed diminished pannus formation when high GA concentrations together with diamine extension were applied (43). Improved tissue fixation through increased cross-link density with resulting surface inflammation quiescence was concomitantly associated with diminished pannus overgrowth. Therefore, not only inflammation with ensuing degradation, nor immune-mediated calcification, but also leaflet stenosis through pannus overgrowth will benefit from insurances of reduced bioprosthetic immunogenicity.

## Rheumatic Heart Disease Analogy

Much of the pathology observed in rheumatic deterioration is analogous to that seen in failed bioprosthetic heart valves. Dystrophic mineralization, stenosis and unresolved chronic inflammation are prominent features of both. Despite it being undisputed that rheumatic heart disease represents the autoimmune consequence of poorly managed rheumatic fever and that the associated calcification is not an inactive process but instead highly regulated through inflammation (77), acknowledgment of a host immune response as the central feature of bioprosthetic pathology, while increasingly undeniable, is seemingly still partially ignored.

## Discussion

**Figure 7:**
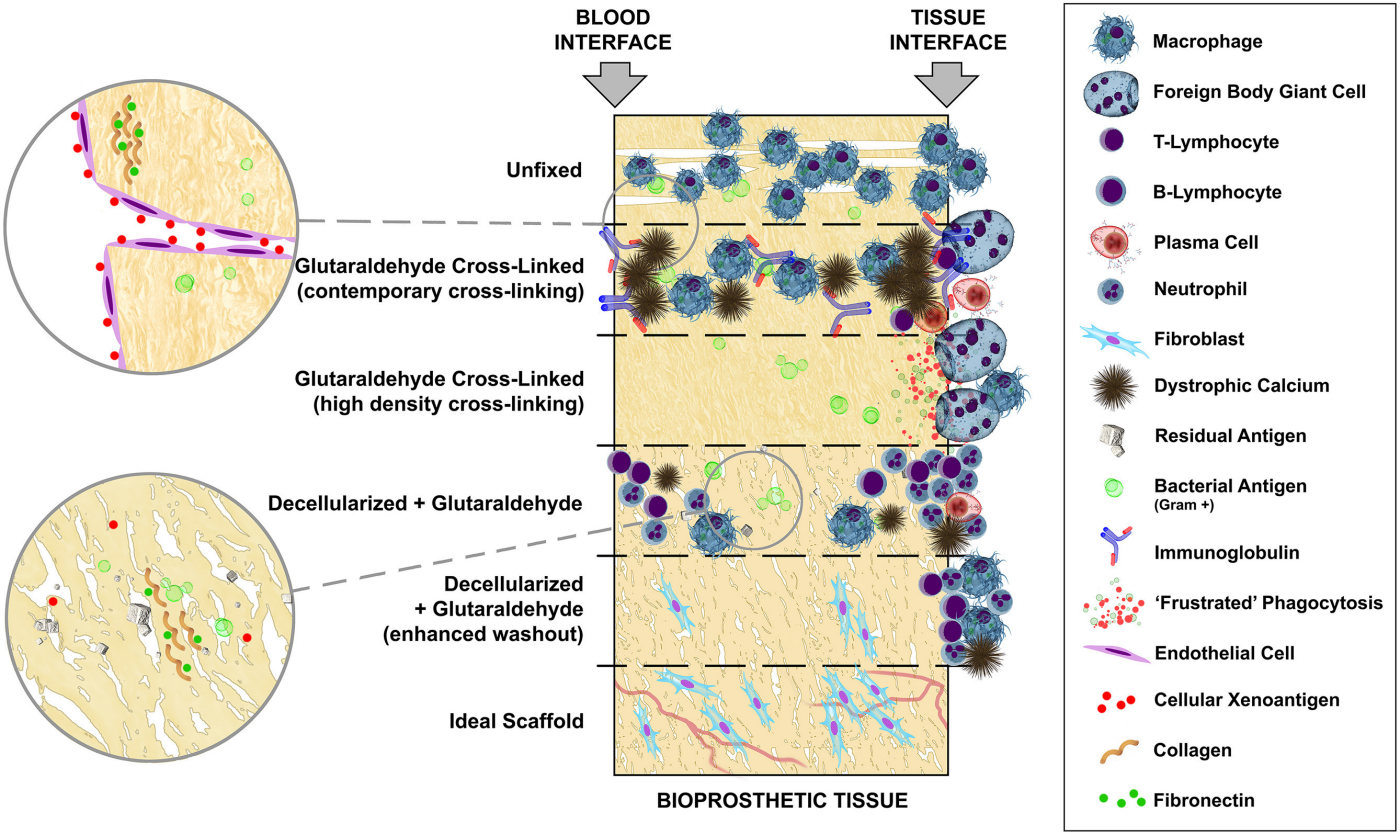
Hypothetical mechanistic overview, based on animal models, of the potential impact of a residual immunogen presence on inflammatory and immune sequelae in bioprosthetic tissue and the ensuing potential for mineralization.

Bioprosthetic replacement heart valves, whether sourced as anatomical porcine aortic valves or fabricated from bovine parietal pericardium are, by definition, xenogeneic and potentially highly immunogenic. If attempts at antigen masking, removal and/or modification are not employed, their implantation in humans should certainly be classified as “discordant.” Limited progress regarding cross-link masking has been made since the first use of GA five decades ago. In addition, the deployment of new and innovative chemistries has been withheld clinically, presumably due to corporate risk assessments including expensive and lengthy regulatory approvals. Antigen removal, through decellularization, may represent a similar concern, which likely stemmed from well-intentioned but failed attempts at the introduction of this technology to leave the door open to repopulation with host tissue by not combining it with antigen masking through cross-linking of the extracellular matrix. Figure 7 provides an overview of the progression of tissue treatments, based on animal studies, from non-crosslinked fresh tissue which undergoes rapid phagocytosis, followed by cross-linking with contemporary GA concentrations incapable of complete antigen masking with its characteristic infiltrating inflammatory and humoral immune response and widespread mineralization. High cross-link density completely removes inflammatory infiltration and tissue-specific immunoglobulin but renders the tissue impractically stiff and subject to “frustrated” phagocytosis with possible latent tissue damage. Decellularization, even with contemporary GA fixation, fails to adequately remove or mask solubilized cellular antigen and introduces problems of microbial remnant antigen (especially when contemporary GA concentrations are used) and other contaminants despite a significant reduction in mineralization. Enhanced passive washout, or via active electrophoresis, of decellularization products may best approach the ideal scaffold, potentially immunologically inert and capable of repopulation and revascularisation.

However, the distinction between cross-species vascularized organ transplantation and the use of largely structural, non-vital tissue used in the replacement of diseased heart valves has become blurred with the consequence that residual immunogenicity of the extracellular matrix has been, unconsciously perhaps, deemed to be inconsequential. Unlike the catastrophic consequences observed in vascularized organs, the pathological response to the bioprosthetic valve is often far more insidious and latent in nature, although can be accelerated in children. The idealistic belief that acellular scaffolds will repopulate and grow with the patient, while desirable, must be tempered with the realization that, while residual immunogens remain, decellularization alone will be inadequate. The growing acceptance of the contribution of an immune-mediated mechanism of dystrophic mineralization together with the potential for pannus-mediated stenosis advocates caution in disregarding the consequences of residual immunogenicity, irrespective of which compartment, cellular or extracellular, that might reside in.

## Author Contributions

PH conceptualized and composed the bulk of the manuscript, it is based on insights acquired through related research performed together with PZ and DB. EA provided valuable insight into inflammatory mechanisms suspected to play a role in the pathology of bioprosthetic heart valves with all three having also intensively reviewed the manuscript. All authors contributed to the article and approved the submitted version.

## Conflict of Interest

The authors declare that the research was conducted in the absence of any commercial or financial relationships that could be construed as a potential conflict of interest.

## Publisher's Note

All claims expressed in this article are solely those of the authors and do not necessarily represent those of their affiliated organizations, or those of the publisher, the editors and the reviewers. Any product that may be evaluated in this article, or claim that may be made by its manufacturer, is not guaranteed or endorsed by the publisher.

## References

[1] Binet JP Carpentier A Langlois J Duran C Colvez P. Implantation de valves heterogenes dans le traitement d e cardiopathies aortiques. C R Acad Hebd Seances Acad Sci D. (1965) 261:5733–4. 4955645

[2] Carpentier A Lemaigre G Robert L Carpentier S Dubost C. Biological factors affecting long-term results of valvular heterografts. J Thorac Cardiovasc Surg. (1969) 58:467–83. 10.1016/S0022-5223(19)42561-0 5344189

[3] Galili U Clark MR Shohet SB Buehler J Macher BA. Evolutionary relationship between the natural anti-Gal antibody and the Gal alpha 1-3Gal epitope in primates. Proc Natl Acad Sci USA. (1987) 84:1369–73. 10.1073/pnas.84.5.1369 2434954 PMC304431

[4] Chen RH Kadner A Mitchell RN Adams DH. Fresh porcine cardiac valves are not rejected in primates. J Thorac Cardiovasc Surg. (2000) 119:1216–20. 10.1067/mtc.2000.106526 10838541

[5] Chen RH Adams DH. Transgenic porcine valves show no signs of delayed cardiac xenograft rejection. Ann Thorac Surg. (2001) 71:S389–92. 10.1016/S0003-4975(01)02505-X 11388231

[6] Van Nooten G Somers P Cornelissen M Bouchez S Gasthuys F Cox E. Acellular porcine and kangaroo aortic valve scaffolds show more intense immune-mediated calcification than cross-linked Toronto SPV valves in the sheep model. Interact Cardiovasc Thorac Surg. (2006) 5:544–9. 10.1510/icvts.2006.136267 17670642

[7] Lila N McGregor CG Carpentier S Rancic J Byrne GW Carpentier A. Gal knockout pig pericardium: new source of material for heart valve bioprostheses. J Heart Lung Transplant. (2010) 29:538–43. 10.1016/j.healun.2009.10.007 20036160

[8] Human P Zilla P. The possible role of immune responses in bioprosthetic heart valve failure. J Heart Valve Dis. (2001) 10:460–6. 11499591

[9] Naso F Gandaglia A Iop L Spina M Gerosa G. First quantitative assay of alpha-Gal in soft tissues: presence and distribution of the epitope before and after cell removal from xenogeneic heart valves. Acta Biomater. (2011) 7:1728–34. 10.1016/j.actbio.2010.11.030 21118731

[10] Galili U LaTemple DC Radic MZ. A sensitive assay for measuring alpha-Gal epitope expression on cells by a monoclonal anti-Gal antibody. Transplantation. (1998) 65:1129–32. 10.1097/00007890-199804270-00020 9583877

[11] Sandrin MS Vaughan HA Dabkowski PL McKenzie IF. Anti-pig IgM antibodies in human serum react predominantly with Gal(alpha 1-3)Gal epitopes. Proc Natl Acad Sci USA. (1993) 90:11391–5. 10.1073/pnas.90.23.11391 7504304 PMC47988

[12] Naso F Gandaglia A Iop L Spina M Gerosa G. Alpha-Gal detectors in xenotransplantation research: a word of caution. Xenotransplantation. (2012) 19:215–20. 10.1111/j.1399-3089.2012.00714.x 22909134

[13] Naso F Gandaglia A Bottio T Tarzia V Nottle MB d'Apice AJ. First quantification of alpha-Gal epitope in current glutaraldehyde-fixed heart valve bioprostheses. Xenotransplantation. (2013) 20:252–61. 10.1111/xen.12044 23865597

[14] Izzat MB Sabbagh N Aljasem H. Early calcific degeneration of the St. Jude Medical Epic aortic bioprosthesis. Clin Case Rep. (2020) 8:387–8. 10.1002/ccr3.2664 32128195 PMC7044358

[15] Moczar M Lecerf L Mazzucotelli JP Loisance D. Immunoglobulins and complement deposits in mitroflow pericardial bioprosthetic heart valves–a contributing factor to structural deterioration. J Heart Valve Dis. (1996) 3:S276–83. 8953454

[16] Zhang R Wang Y Chen L Wang R Li C Li X. Reducing immunoreactivity of porcine bioprosthetic heart valves by genetically-deleting three major glycan antigens, GGTA1/beta4GalNT2/CMAH. Acta Biomater. (2018) 72:196–205. 10.1016/j.actbio.2018.03.055 29631050

[17] Salama A Evanno G Harb J Soulillou JP. Potential deleterious role of anti-Neu5Gc antibodies in xenotransplantation. Xenotransplantation. (2015) 22:85–94. 10.1111/xen.12142 25308416

[18] Reuven EM Leviatan Ben-Arye S Marshanski T Breimer ME Yu H Fellah-Hebia I. Characterization of immunogenic Neu5Gc in bioprosthetic heart valves. Xenotransplantation. (2016) 23:381–92. 10.1111/xen.12260 27610947 PMC5036590

[19] Lee W Hara H Ezzelarab MB Iwase H Bottino R Long C. Initial *in vitro* studies on tissues and cells from GTKO/CD46/NeuGcKO pigs. Xenotransplantation. (2016) 23:137–50. 10.1111/xen.12229 26988899 PMC4842123

[20] Lee W Long C Ramsoondar J Ayares D Cooper DK Manji RA. Human antibody recognition of xenogeneic antigens (NeuGc and Gal) on porcine heart valves: could genetically modified pig heart valves reduce structural valve deterioration? Xenotransplantation. (2016) 23:370–80. 10.1111/xen.12254 27511593

[21] Naso F Stefanelli U Buratto E Lazzari G Perota A Galli C. Alpha-gal inactivated heart valve bioprostheses exhibit an anti-calcification propensity similar to knockout tissues < sup/>. Tissue Eng Part A. (2017) 23:1181–95. 10.1089/ten.tea.2016.0474 29053434

[22] LaVecchio JA Dunne AD Edge ASB. Enzymatic removal of alpha-galactosyl epitopes from porcine endothelial cells diminishes the cytotoxic effect of natural antibodies. Transplantation. (1995) 60:841–7. 10.1097/00007890-199510270-00014 7482745

[23] Park S Kim WH Choi SY Kim YJ. Removal of alpha-Gal epitopes from porcine aortic valve and pericardium using recombinant human alpha galactosidase A. J Korean Med Sci. (2009) 24:1126–31. 10.3346/jkms.2009.24.6.1126 19949670 PMC2775862

[24] Kim MS Lim HG Kim YJ. Calcification of decellularized and alpha-galactosidase-treated bovine pericardial tissue in an alpha-Gal knock-out mouse implantation model: comparison with primate pericardial tissue. Eur J Cardiothorac Surg. (2016) 49:894–900. 10.1093/ejcts/ezv189 25994817

[25] Schmoeckel M Nollert G Shahmohammadi M Muller-Hocker J Young VK Kasper-Konig W. Transgenic human decay accelerating factor makes normal pigs function as a concordant species. J Heart Lung Transplant. (1997) 16:758–64. 9257258

[26] Manji RA Ekser B Menkis AH Cooper DK. Bioprosthetic heart valves of the future. Xenotransplantation. (2014) 21:1–10. 10.1111/xen.12080 24444036 PMC4890621

[27] Manji RA Lee W Cooper DKC. Xenograft bioprosthetic heart valves: Past, present and future. Int J Surg. (2015) 23:280–4. 10.1016/j.ijsu.2015.07.009 26190838

[28] Zilla P Zhang Y Human P Koen W vonOppell U.. Improved ultrastructural preservation of bioprosthetic tissue. J Heart Valve Dis. (1997) 6:492–501. 9330171

[29] Human P Weissenstein C Trantina A Zilla P. Fixation-related autolysis and bioprosthetic aortic wall calcification. J Heart Valve Dis. (2001) 10:656−65. 11603606

[30] Ueda Y Torrianni MW Coppin CM Iwai S Sawa Y Matsuda H. Antigen clearing from porcine heart valves with preservation of structural integrity. Int J Artif Organs. (2006) 29:781–9. 10.1177/039139880602900808 16969756

[31] Paniagua Gutierrez JR Berry H Korossis S Mirsadraee S Lopes SV da Costa F. Regenerative potential of low-concentration SDS-decellularized porcine aortic valved conduits in vivo. Tissue Eng Part A. (2015) 21:332–42. 10.1089/ten.tea.2014.0003 25156153 PMC4293138

[32] Iwai S Torikai K Coppin CM Sawa Y. Minimally immunogenic decellularized porcine valve provides in situ recellularization as a stentless bioprosthetic valve. J Artif Organs. (2007) 10:29–35. 10.1007/s10047-006-0360-1 17380294

[33] Grebenik EA Gafarova ER Istranov LP Istranova EV Ma X Xu J. Mammalian pericardium-based bioprosthetic materials in xenotransplantation and tissue engineering. Biotechnol J. (2020) 15:e1900334. 10.1002/biot.201900334 32077589

[34] Simon P Kasimir MT Seebacher G Weigel G Ullrich R Salzer-Muhar U. Early failure of the tissue engineered porcine heart valve SYNERGRAFT in pediatric patients. Eur J Cardiothorac Surg. (2003) 23:1002–6. 10.1016/S1010-7940(03)00094-0 12829079

[35] Cicha I Ruffer A Cesnjevar R Glockler M Agaimy A Daniel WG. Early obstruction of decellularized xenogenic valves in pediatric patients: involvement of inflammatory and fibroproliferative processes. Cardiovasc Pathol. (2011) 20:222–31. 10.1016/j.carpath.2010.04.006 20598910

[36] Naso F Gandaglia A. Different approaches to heart valve decellularization: A comprehensive overview of the past 30 years. Xenotransplantation. (2018) 25:12354. 10.1111/xen.12354 29057501

[37] Liu X Li N Gong D Xia C Xu Z. Comparison of detergent-based decellularization protocols for the removal of antigenic cellular components in porcine aortic valve. Xenotransplantation. (2018) 25:e12380. 10.1111/xen.12380 29446183

[38] Booth C Korossis SA Wilcox HE Watterson KG Kearney JN Fisher J. Tissue engineering of cardiac valve prostheses I: development and histological characterization of an acellular porcine scaffold. J Heart Valve Dis. (2002) 11:457–62. 12150290

[39] Luo Y Ma L. Bioprosthetic heart valves with reduced immunogenic residuals using vacuum-assisted decellularization treatment. Biomed Mater. (2020) 15:065012. 10.1088/1748-605X/abaabf 33016260

[40] Duran CJ. Tissue Electrophoresis for Generation of Porcine Acellular Dermal Matrices [dissertation/master's thesis]. Colorado State University (2013).

[41] Arai S Lacerda C Orton EC. Tissue-gel electrophoresis enhances antigen removal from porcine aortic valve and bovine pericardium. J Heart Valve Dis. (2010) 19:753–8. 21214100

[42] Wong ML Leach JK Athanasiou KA Griffiths LG. The role of protein solubilization in antigen removal from xenogeneic tissue for heart valve tissue engineering. Biomaterials. (2011) 32:8129–38. 10.1016/j.biomaterials.2011.07.030 21810537

[43] Human P Zilla P. Inflammatory and immune processes: the neglected villain of bioprosthetic degeneration? J Long Term Eff Med Implants. (2001) 11:199–220. 10.1615/JLongTermEffMedImplants.v11.i34.80 11921664

[44] Human P Ofoegbu C Ilsley H Bezuidenhout D de Villiers J Williams DF. Decellularization and engineered crosslinking: a promising dual approach towards bioprosthetic heart valve longevity. Eur J Cardiothorac Surg. (2020) 58:1192–200. 10.1093/ejcts/ezaa257 32893300

[45] Human P Zilla P. Characterization of the immune response to valve bioprostheses and its role in primary tissue failure. Ann Thorac Surg. (2001) 71:S385–8. 10.1016/S0003-4975(01)02492-4 11388230

[46] Mirsadraee S Wilcox HE Watterson KG Kearney JN Hunt J Fisher J. Biocompatibility of acellular human pericardium. J Surg Res. (2007) 143:407–14. 10.1016/j.jss.2007.01.026 17574597

[47] Bayrak A Pruger P Stock UA Seifert M. Absence of immune responses with xenogeneic collagen and elastin. Tissue Eng Part A. (2013) 19:1592–600. 10.1089/ten.tea.2012.0394 23406399 PMC3665304

[48] Biermann AC Marzi J Brauchle E Schneider M Kornberger A Abdelaziz S. Impact of T-cell-mediated immune response on xenogeneic heart valve transplantation: short-term success and mid-term failure. Eur J Cardiothorac Surg. (2018) 53:784–92. 10.1093/ejcts/ezx396 29186380 PMC5848813

[49] Simon P Kasimir MT Rieder E Weigel G. Tissue Engineering of heart valves-Immunologic and inflammatory challenges of the allograft scaffold. Progress Pediatric Cardiol. (2006) 21:161–5. 10.1016/j.ppedcard.2005.11.004

[50] Hinderer S Schesny M Bayrak A Ibold B Hampel M Walles T. Engineering of fibrillar decorin matrices for a tissue-engineered trachea. Biomaterials. (2012) 33:5259–66. 10.1016/j.biomaterials.2012.03.075 22521489

[51] Liu Z Wang Y Xie F Liu X Li F Dong N. Elimination of macrophages reduces glutaraldehyde-fixed porcine heart valve degeneration in mice subdermal model. Pharmacol Res Perspect. (2021) 9:e00716. 10.1002/prp2.716 33523576 PMC7849454

[52] Blitzer D Yedlicka G Manghelli J Dentel J Caldwell R Brown JW. Twin-to-twin heart transplantation: a unique event with a 25-year follow-up. Ann Thorac Surg. (2017) 103:e341–2. 10.1016/j.athoracsur.2016.09.060 28359493

[53] Lim WH Gray NA Chadban SJ Pilmore H Wong G. Graft and patient outcomes of zero-human leucocyte-antigen-mismatched deceased and live donor kidney transplant recipients. Transpl Int. (2015) 28:610–8. 10.1111/tri.12542 25689280

[54] Krishnan N Buchanan PM Dzebisashvili N Xiao H Schnitzler MA Brennan DC. Monozygotic transplantation: concerns and opportunities. Am J Transplant. (2008) 8:2343–51. 10.1111/j.1600-6143.2008.02378.x 18808409 PMC2678894

[55] Kanzaki T Yamagishi M Yashima M Yaku H. Seven-year outcome of pulmonary valve autograft replacement of the mitral valve in an infant. J Thorac Cardiovasc Surg. (2011) 141:e33–5. 10.1016/j.jtcvs.2011.01.049 21377700

[56] El-Hamamsy I Eryigit Z Stevens LM Sarang Z George R Clark L. Long-term outcomes after autograft versus homograft aortic root replacement in adults with aortic valve disease: a randomised controlled trial. Lancet. (2010) 376:524–31. 10.1016/S0140-6736(10)60828-8 20684981

[57] Mookhoek A de Heer E Bogers AJ Takkenberg JJ Schoof PH. Pulmonary autograft valve explants show typical degeneration. J Thorac Cardiovasc Surg. (2010) 139:1416–9. 10.1016/j.jtcvs.2010.01.020 20363479

[58] Rabkin-Aikawa E Aikawa M Farber M Kratz JR Garcia-Cardena G Kouchoukos NT. Clinical pulmonary autograft valves: pathologic evidence of adaptive remodeling in the aortic site. J Thorac Cardiovasc Surg. (2004) 128:552–61. 10.1016/j.jtcvs.2004.04.016 15457156

[59] Dignan R O'Brien M Hogan P Passage J Stephens F Thornton A. Influence of HLA matching and associated factors on aortic valve homograft function. J Heart Valve Dis. (2000) 9:504–11. 10947042

[60] Legare JF Lee TD Creaser K Ross DB. T lymphocytes mediate leaflet destruction and allograft aortic valve failure in rats. Ann Thorac Surg. (2000) 70:1238–45. 10.1016/S0003-4975(00)01677-5 11081878

[61] Rajani B Mee RB Ratliff NB. Evidence for rejection of homograft cardiac valves in infants. J Thorac Cardiovasc Surg. (1998) 115:111–7. 10.1016/S0022-5223(98)70449-0 9451053

[62] Eishi K Ishibashi-Ueda H Nakano K Kosakai Y Sasako Y Kobayashi J. Calcific degeneration of bioprosthetic aortic valves in patients receiving steroid therapy. J Heart Valve Dis. (1996) 5:668–72. 8953446

[63] Calne RY. Organ transplantation between widely disparate species. Transplant Proc. (1970) 2:550–6. 10.1136/bmj.2.5701.111-a 5000236

[64] Rose AG Cooper DK Human PA Reichenspurner H Reichart B. Histopathology of hyperacute rejection of the heart: experimental and clinical observations in allografts and xenografts. J Heart Lung Transplant. (1991) 10:223–34. 2031919

[65] Cooper DK Human PA Lexer G Rose AG Rees J Keraan M. Effects of cyclosporine and antibody adsorption on pig cardiac xenograft survival in the baboon. J Heart Transplant. (1988) 7:238–46. 3290407

[66] Carpentier SM Monier MH Shen M Carpentier AF. Do donor or recipient species influence calcification of bioprosthetic tissues? Ann Thorac Surg. (1995) 60:S328–30. 10.1016/0003-4975(95)00244-F 7646182

[67] Grabenwoger M Grimm M Eybl E Kadletz M Havel M Kostler P. New aspects of the degeneration of bioprosthetic heart valves after long-term implantation. J Thorac Cardiovasc Surg. (1992) 104:14–21. 10.1016/S0022-5223(19)34831-7 1614200

[68] Jeong S Yoon EJ Lim HG Sung SC Kim YJ. The effect of space fillers in the cross-linking processes of bioprosthesis. Biores Open Access. (2013) 2:98–106. 10.1089/biores.2012.0289 23593562 PMC3620541

[69] Somers P De Somer F Cornelissen M Bouchez S Gasthuys F Narine K. Genipin blues: an alternative non-toxic crosslinker for heart valves? J Heart Valve Dis. (2008) 17:682–8. 19137802

[70] Fiebig U Fischer K Bahr A Runge C Schnieke A Wolf E. Porcine endogenous retroviruses: Quantification of the copy number in cell lines, pig breeds, and organs. Xenotransplantation. (2018) 25:e12445. 10.1111/xen.12445 30264881

[71] Jensen HE Gyllensten J Hofman C Leifsson PS Agerholm JS Boye M. Histologic and bacteriologic findings in valvular endocarditis of slaughter-age pigs. J Vet Diagn Invest. (2010) 22:921–7. 10.1177/104063871002200611 21088176

[72] Arellano CK. The Calcification of Staphylococcus aureus Bacteria: A Potential Defense Mechanism Against Infections [dissertation/master's thesis]. University of California, San Diego (2010).

[73] Truong LY. The Calcification of Staphylococcus aureus Bacteria by the Mineralization by Inhibitor Exclusion Mechanism: A Potential Defense Mechanism Against Bacterial Infections [dissertation/master's thesis]. University of California, San Diego (2011).

[74] Koo HJ Choe J Kang DY Ko E Ahn JM Park DW. Computed tomography features of cuspal thrombosis and subvalvular tissue ingrowth after transcatheter aortic valve implantation. Am J Cardiol. (2020) 125:597–606. 10.1016/j.amjcard.2019.11.015 31839148

[75] Greisler HP Dennis JW Endean ED Ellinger J Friesel R Burgess W. Macrophage/biomaterial interactions: the stimulation of endothelialization. J Vasc Surg. (1989) 9:588–93. 10.1016/0741-5214(89)90478-3 2523491

[76] Wewers MD Marsh CB. Role of the antibody in the pathogenesis of transplant vascular sclerosis: a hypothesis. Transpl Immunol. (1997) 5:283–8. 10.1016/S0966-3274(97)80009-3 9504148

[77] Chopra P Gulwani H. Pathology and pathogenesis of rheumatic heart disease. Indian J Pathol Microbiol. (2007) 50:685–97. 18306530

